# Parsimonious machine learning models to predict resource use in cardiac surgery across a statewide collaborative

**DOI:** 10.1016/j.xjon.2022.04.017

**Published:** 2022-04-20

**Authors:** Arjun Verma, Yas Sanaiha, Joseph Hadaya, Anthony Jason Maltagliati, Zachary Tran, Ramin Ramezani, Richard J. Shemin, Peyman Benharash, the University of California Cardiac Surgery Consortium, Peyman Benharash, Richard J. Shemin, Nancy Satou, Tom Nguyen, Carolyn Clary, Michael Madani, Jill Higgins, Dawna Steltzner, Bob Kiaii, J. Nilas Young, Kathleen Behan, Heather Houston, Cindi Matsumoto, Jack C. Sun, Lisha Flavin, Patria Fopiano, Maricel Cabrera, Rakan Khaki, Polly Washabaugh

**Affiliations:** a Cardiovascular Outcomes Research Laboratories, University of California Los Angeles, Los Angeles, Calif; b Department of Surgery, Harbor-UCLA Medical Center, Los Angeles, Calif; c Department of Computer Science, University of California Los Angeles, Los Angeles, Calif; d Division of Cardiac Surgery, University of California Los Angeles, Los Angeles, Calif

**Keywords:** cardiac surgery, resource utilization, length of stay, machine learning, COVID-19, AKI, acute kidney injury, AUC, area under the receiver operating characteristic, CABG, coronary artery bypass grafting, GBM, gradient boosted machine, ICU, intensive care unit, LOS, length of stay, ML, machine learning, RF, random forest, STS, Society of Thoracic Surgeons, UCCSC, University of California Cardiac Surgery Consortium, XGBoost, extreme gradient boosting

## Abstract

**Objective:**

We sought to several develop parsimonious machine learning models to predict resource utilization and clinical outcomes following cardiac operations using only preoperative factors.

**Methods:**

All patients undergoing coronary artery bypass grafting and/or valve operations were identified in the 2015-2021 University of California Cardiac Surgery Consortium repository. The primary end point of the study was length of stay (LOS). Secondary endpoints included 30-day mortality, acute kidney injury, reoperation, postoperative blood transfusion and duration of intensive care unit admission (ICU LOS). Linear regression, gradient boosted machines, random forest, extreme gradient boosting predictive models were developed. The coefficient of determination and area under the receiver operating characteristic (AUC) were used to compare models. Important predictors of increased resource use were identified using SHapley summary plots.

**Results:**

Compared with all other modeling strategies, gradient boosted machines demonstrated the greatest performance in the prediction of LOS (coefficient of determination, 0.42), ICU LOS (coefficient of determination, 0.23) and 30-day mortality (AUC, 0.69). Advancing age, reduced hematocrit, and multiple-valve procedures were associated with increased LOS and ICU LOS. Furthermore, the gradient boosted machine model best predicted acute kidney injury (AUC, 0.76), whereas random forest exhibited greatest discrimination in the prediction of postoperative transfusion (AUC, 0.73). We observed no difference in performance between modeling strategies for reoperation (AUC, 0.80).

**Conclusions:**

Our findings affirm the utility of machine learning in the estimation of resource use and clinical outcomes following cardiac operations. We identified several risk factors associated with increased resource use, which may be used to guide case scheduling in times of limited hospital capacity.

## Graphical abstract

**Figure:**
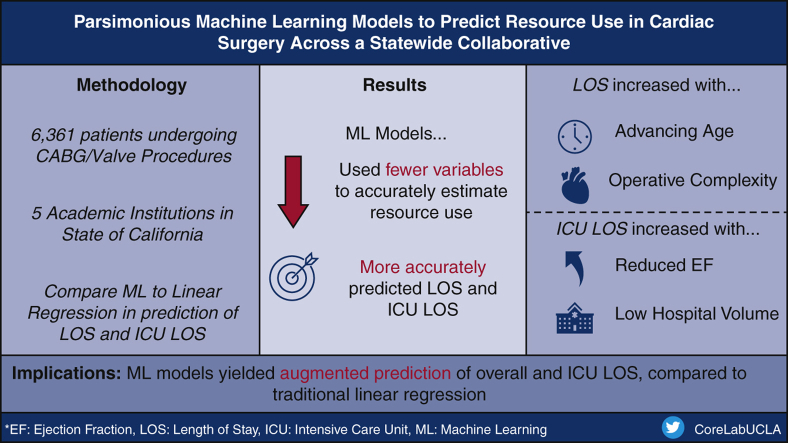

**Figure:**
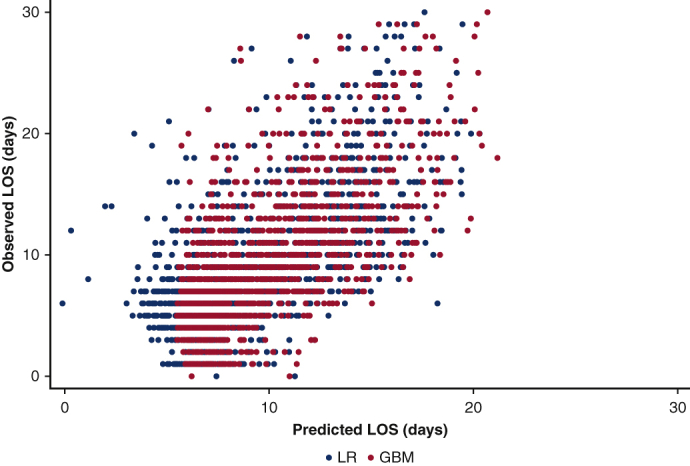
Observed length of stay versus predictions by machine learning model.

**Central Message**

Compared to traditional linear regression, machine learning yielded superior performance in the prediction of length of stay, mortality, acute kidney injury, and reoperation following cardiac operations.

**Perspective**

This study outlined the development of machine learning (ML) models to predict length of stay (LOS) following cardiac operations. Several clinical, operation-related, and hospital characteristics were found to be associated with increased LOS. Taken together, our findings suggest that ML models may be used to inform case scheduling strategies during times of limited hospital capacity.

The novel COVID-19 pandemic has placed unprecedented strain on health care systems, influencing the allocation of personnel and resources. Several groups have reported cardiac surgery case volume reductions of 45% to 94%, with significant regional variability.^1^, ^2^, ^3^, ^4^ Subject to rates of “reopening” and patients’ desire to proceed with elective surgery, the projected time to equilibrium between back-logged cases and ongoing surgical need is estimated to be 12 to 22 months.^5^, ^6^, ^7^ Furthermore, recovery from cessation of elective cases requires a nuanced approach to management of deferred and newly presenting patients as well as ongoing demands for perioperative resources. With estimates that operating volume must exceed 120% of baseline to accommodate deferred patients while concurrently preventing excess waitlist morbidity, rapid and accurate prediction of hospital bed occupancy and resource utilization are especially crucial.^6^

The Society of Thoracic Surgeons (STS), among others, has successfully implemented risk models to provide canonical estimates for parameters such as mortality, postoperative complications, and prolonged length of stay (LOS).^8^ However, as demonstrated by several reports of poor calibration when applied at the institutional level, these predictive tools are often cumbersome and require numerous data fields to yield a predicted risk without accounting for local variations in clinical practice.^9^, ^10^, ^11^ Furthermore, most available models predict prolonged LOS in a binary manner, rather than an estimate of the actual duration of hospitalization in days.^8^^,^^12^, ^13^, ^14^ The classification of LOS into prolonged and routine reduces generalizability and limits the application of such tools in acute care settings.^15^

Machine learning (ML) algorithms allow for complex modeling of nonlinear relationships between predictive factors and have demonstrated superior discrimination and calibration in several clinical applications.^16^, ^17^, ^18^ Therefore, we sought to develop ML-based models to predict LOS, 30-day mortality and select complications using an academic, statewide database. We hypothesized that a parsimonious ML model, containing few explanatory covariates, would yield superior discrimination and calibration compared with traditional linear and logistic regression.

## Methods

### Study Population

All adults (aged 18 years or older) who underwent coronary artery bypass grafting (CABG) and/or valve operations were identified from the 2015 to 2021 University of California Cardiac Surgery Consortium (UCCSC) repository. Founded in 2013, the UCCSC is a collaborative among 5 academic hospitals across California. Data elements, including those submitted to the STS, are prospectively collected in compliance with policies of individual institutions and the University of California Systemwide Review Board (IRB No. 16-000558, approved May 6, 2016, renewed April 15, 2020). The need for patient written consent for the publication of the study data was waived by the institutional review board due to the de-identified nature of the UCCSC.

**Figure E1:**
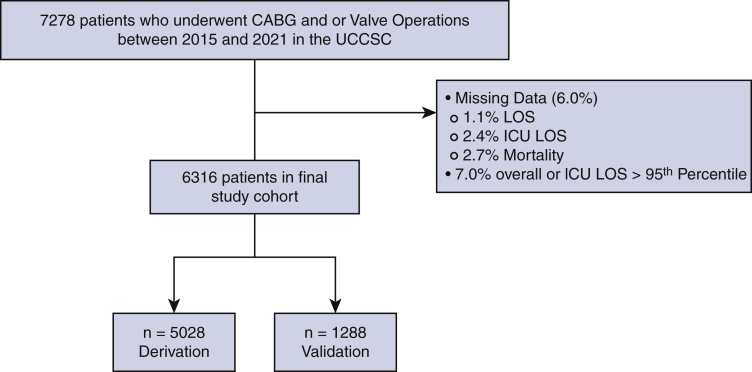
Study Consolidated Standards of Reporting Trials diagram. *CABG*, Coronary artery bypass grafting; *UCCSC*, University of California Cardiac Surgery Consortium; *LOS*, length of stay; *ICU LOS*, intensive care unit length of stay.

Patients were stratified by the class of operation performed: isolated CABG, isolated valve, concomitant CABG/valve and multivalve operations. Those who required left ventricular assist device implantation, extracorporeal membrane oxygenation, or transcatheter procedures were excluded to maintain cohort homogeneity. Moreover, records with missing values for overall and intensive care unit (ICU) LOS as well as 30-day mortality were excluded (Figure E1). Patients with LOS or ICU LOS >95th percentile (>30 days for LOS, >259 hours in ICU) were similarly excluded.

### Variable and Outcome Definitions

The primary end point was overall LOS. Mortality at 30 days, acute kidney injury (AKI), postoperative blood transfusion, reoperation and ICU LOS were also considered. Patient comorbidities, operative characteristics, and complications including AKI, postoperative blood transfusion and reoperation, were defined in accordance with the STS Adult Cardiac Database dictionary.^19^ Annual operative caseload, number of adult cardiac surgeons, total number of low acuity and cardiothoracic ICU beds were tabulated for each institution. Variables with missing values in >20% of patients were not considered for inclusion. For retained features with missing data, values were imputed using the median and mode for continuous and categorical variables, respectively. The number of records with missing data for each variable is reported in Table E1.

### Modeling Techniques

**Figure E2:**
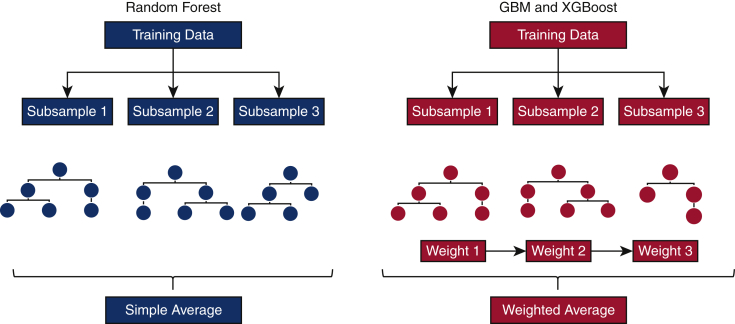
Schematic representing the algorithmic design of random forest, gradient boosted machines (*GBM*) and extreme gradient boosting (*XGBoost*).

We compared 3 ML algorithms to traditional, multivariable linear, and logistic regression: gradient boosted machines (GBM), extreme gradient boosting (XGBoost) and random forest (RF). These algorithms autonomously generate a large set of decision trees to capture nuanced patterns in training data. In the case of RF, the development of every decision tree occurs independently, and the final output of the model is the arithmetic mean of the output from each decision tree. However, the XGBoost and GBM algorithms train decision trees in a stepwise manner to compensate for errors of the prior trees, and the output is the weighted average of each decision tree's estimate.^20^ A brief schematic highlighting the differences between boosting (XGBoost and GBM) and bagging (RF) classifiers is shown in Figure E2. Hyperparameters, which are used to control the learning process of ML models, were selected using the GridSearchCV function in the Python *sklearn* library (Python Software Foundation). This technique exhaustively evaluates a wide range of hyperparameters and selects values that optimize model performance. Selected hyperparameters for each model are shown in Table E2.

### Model Development

Thirty-seven preoperative patient and hospital characteristics were chosen as candidate predictors. Clinical variables were selected from the STS risk score variable list based on clinical relevance and are listed in Table 1.^8^ Hospital factors were incorporated to account for variation in practice across participating institutions. Variable selection was performed using recursive feature elimination, a ML technique that is used to reduce collinearity and eliminate covariates with low variance. In recursive feature elimination, cross-validation is used to exhaustively evaluate variable sets of different sizes and select the best collection of features. Given that transportability and ease of use is an important aspect of risk tools, we identified the smallest set of variables that retained maximum predictive performance. This algorithm was independently applied using linear regression and GBM to ascertain any differences between modeling strategies. Selected variables were used for all subsequent model development (Table E3). We also compared the performance of ML against the STS risk scores for 30-day mortality, AKI, and reoperation.

**Table 1:** Baseline patient characteristics of the study cohort

Parameter	Overall (n = 6316)	Derivation (n = 5028)	Validation (n = 1288)	*P* value
Age (y)	63 ± 13	63 ± 13	64 ± 13	<.001
Elective admission (%)	58.5	58.3	59.3	.52
Female (%)	27.5	27.7	26.6	.45
Height (cm)	171 ± 11	171 ± 11	171 ± 10	.29
Weight (kg)	82 ± 19	82 ± 19	81 ± 20	.57
Ethnicity (%)	19.7	19.9	19.3	.68
Operative type (%)				
Isolated CABG	50.5	51.3	47.4	.012
Isolated valve operation	33.3	31.3	41.2	<.001
CABG + valve	10.6	11.1	8.3	.003
Multiple valve	5.8	6.3	3.6	<.001
Medical conditions (%)				
Atrial fibrillation	17.6	17.6	17.7	.91
Cancer	6.9	7.1	6.4	.37
Cerebrovascular disease	17.0	17.2	16.2	.38
Severe lung disease	3.3	3.2	3.9	.23
Congestive heart failure	36.1	33.8	45.0	<.001
Diabetes	38.1	37.6	39.7	.18
Home oxygen	3.0	3.1	2.8	.59
Hypertension	77.4	77.2	78.0	.57
Infectious endocarditis	5.9	5.9	6.1	.84
Liver disease	6.4	6.8	5.0	.017
Peripheral vascular disease	9.0	8.5	11.1	.003
Prior myocardial infarction	31.2	31.7	29.3	.09
Laboratory values				
Hematocrit (% blood volume)	39 ± 6	39 ± 6	39 ± 6	.01
International normalized ratio	1.13 ± 0.3	1.13 ± 0.3	1.12 ± 0.2	.26
Serum albumin (g/dL)	3.9 ± 0.6	3.9 ± 0.6	3.9 ± 0.6	.008
Preoperative creatinine (mg/dL)	1.4 ± 1.7	1.4 ± 1.6	1.5 ± 1.9	<.001
Ejection fraction (%)	56 ± 12	56 ± 12	57 ± 12	.21
Hospital of operation (%)				
Center 1	32.7	31.7	36.5	.001
Center 2	24.0	24.7	21.4	.014
Center 3	19.1	19.1	18.9	.84
Center 4	14.0	14.9	10.5	<.001
Center 5	10.2	9.5	12.7	<.001

Values are presented as mean ± SD or n. *CABG*, Coronary artery bypass grafting.

The derivation cohort consisted of operations performed before March 2020, whereas the remainder comprised the validation dataset. To obtain cross-validated performance metrics, models were fit using 50% of the derivation cohort and tested using the remainder. This process was repeated 100 times to acquire model performance metrics, which are reported as means with 95% CIs. To account for potential differences in case-mix due to the COVID-19 pandemic, we assessed the stability of model performance in the pre- (derivation) and post-COVID–19 (validation) eras.

### Model Evaluation and Interpretation

Linear regression, GBM, RF, and XGBoost models were compared using the coefficient of determination (*R*^2^) between observed and predicted values. Binary classifiers were evaluated using the area under the receiver operating characteristic (AUC). The accuracy of probabilistic predictions was assessed using the Brier score, for which lower values denote superior calibration. Model *R*^2^ and Brier scores were analyzed using a paired *t* test, which allowed for comparison of model performance across cross-validation folds. Similarly, model AUCs were compared using DeLong's test, which specifically accounts for the influence of model evaluation on a common test set. SHapley additive values were calculated to estimate the marginal influence of each covariate on the output of a decision tree model.^17^

Baseline characteristics are reported as means with SD or medians with interquartile range (IQR), as appropriate. Means were analyzed using the adjusted Wald test, whereas medians were analyzed with the Mann-Whitney *U* test. Categorical variables are reported as frequencies and were compared using the Pearson χ^2^ test. Statistical significance was set at α = 0.05. Statistical analysis was conducted using Stata 16.0 (StataCorp) and Python version 3.9. The *sklearn, shap*, and *xgboost* packages of Python were used to develop and assess ML models as described above.^21^^,^^22^

## Results

### Population Characteristics

Across 5 participating centers, 6,316 patients met study criteria. The study cohort was predominantly male (72.5%), with mean age 63 years. A significant proportion of patients had preexisting medical conditions such as diabetes, congestive heart failure, and atrial fibrillation (Table 1). The most frequent operation was isolated CABG (50.5%), followed by isolated valve (33.3%) and concomitant CABG/valve operations (10.6%). The majority of operations were performed electively. Over the study period, the highest volume center performed 1,205 operations, whereas the lowest volume center performed 626 operations. The 30-day mortality rate was 0.9%. Overall, 27.7% of patients received postoperative transfusions, and 1.5% developed AKI. Median LOS was 8 days (IQR, 6-13 days) with a median ICU LOS of 74 hours (IQR, 47-116 hours).

Comparison of baseline characteristics and outcomes between the derivation and validation cohorts is shown in Tables 1 and 2. Patients in the validation cohort were marginally older (64 ± 13 vs 63 ± 13 years; *P* <.001) and had greater rates of congestive heart failure (45.0% vs 33.8%; *P* < .001) and peripheral vascular disease (11.1% vs 8.3%; *P* = .003). Valve operations were more frequent in the validation group, compared with derivation. Although rates of 30-day mortality and AKI were similar, the incidence of reoperation (6.9% vs 9.1%; *P* = .014) and postoperative blood transfusion (23.1% vs 28.8%; *P* < .001) was lower in the validation cohort. The distribution of LOS and ICU LOS was statistically different between the derivation and validation datasets (Table 2).

**Table 2:** Resource utilization and clinical outcomes stratified by derivation and validation cohorts

Outcome	Overall (n = 6316)	Derivation (n = 5028)	Validation (n = 1288)	*P* value
Resource use				
Length of stay (d)	8 (6-13)	8 (6-13)	8 (5-12)	.008
ICU length of stay (h)	74 (47-116)	75 (47-117)	68 (43-99)	<.001
Clinical end points				
Mortality	0.9	1.0	0.7	.39
Acute kidney injury	1.5	1.5	1.7	.54
Postoperative transfusion	27.7	28.8	23.1	<.001
Reoperation	8.6	9.1	6.9	.014

Values are presented as median (interquartile range) or %. *ICU*, Intensive care unit.

### Variable Selection

**Figure 1:**
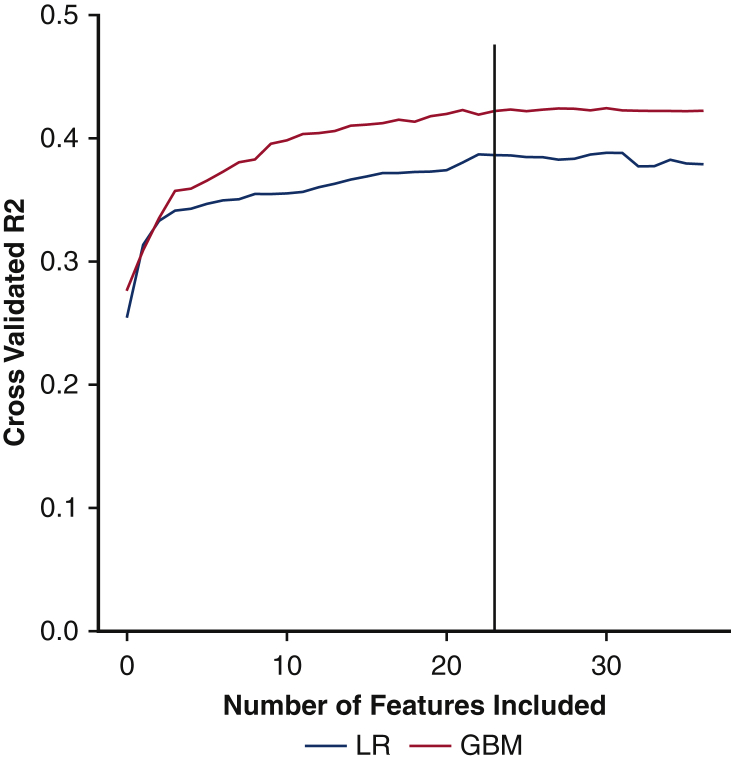
Coefficient of determination (R^2^) versus covariate set size in the prediction of in-hospital length of stay. *LR*, Linear regression; *GBM*, gradient boosted machine.

Recursive feature elimination was applied to 37 candidate variables to determine the optimal covariate set in the prediction of overall LOS. Figure 1 demonstrates the cross-validated *R*^2^ versus the number of covariates included in each model. The GBM model outperformed linear regression, regardless of feature set size. Notably, after the inclusion of 23 features, no appreciable increase in performance was observed from the GBM or linear regression model. Thus, all models were developed using the 23 features that were most strongly associated with LOS (Table E3).

### Resource Utilization

**Figure E3:**
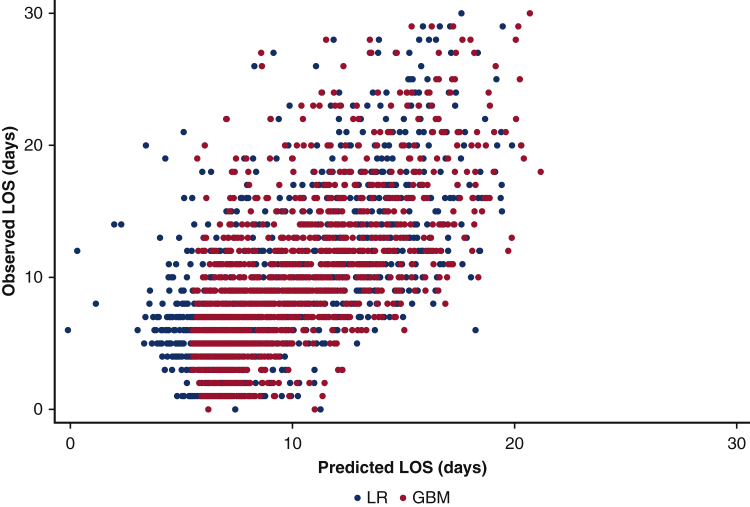
Calibration plot of observed versus predicted length of stay in days. *R*^*2*^, Coefficient of determination; *GBM*, gradient boosted machine; *LR*, Linear regression.

Linear regression, GBM, RF, and XGBoost models were developed to predict in-hospital LOS. Compared to linear regression, the GBM model yielded a higher *R*^2^ (0.42 vs 0.41; *P* < .001, Table 3). As shown in Figure E3, predictions by the GBM model were more strongly correlated with observed values for LOS, compared with linear regression. Although the difference in cross-validated *R*^2^ between the 2 strategies was subtle, the GBM model greatly outperformed linear regression in the validation dataset (*R*^2^, 0.47 vs 0.42) (Table 4). When assessing cumulative model error in the validation cohort, the GBM model resulted in a 197-day reduction in error across all patients relative to linear regression.

**Table 3:** Performance of each algorithm when predicting resource utilization and clinical outcomes in the validation cohort

Outcome	Linear	Logistic	GBM	RF	XGBoost	STS
Resource use∗						–
Length of stay	0.42	–	0.47	0.47	0.47	–
ICU length of stay	0.017	–	0.078	0.054	0.082	
Clinical end point†						
Mortality	–	0.68	0.68	0.7	0.72	0.91
Acute kidney injury	–	0.77	0.79	0.8	0.8	0.84
Postoperative transfusion	–	0.69	0.68	0.68	0.67	–
Reoperation	–	0.78	0.79	0.8	0.78	0.76

*GBM*, Gradient boosted machine; *RF*, random forest; *XGBoost*, extreme gradient boosting; *STS*, Society of Thoracic Surgeons risk score; *ICU*, intensive care unit.

∗ Regressions were evaluated using the coefficient of determination (R2).

† Binary classifiers were assessed with the area under the receiver operating characteristic.

**Table 4:** Cross-validated model performance metrics for each algorithm and outcome

Outcome	Linear	Logistic	GBM	RF	XGBoost	STS	*P* value	*P* value
Resource use∗								
Length of stay	0.41 (0.41-0.41)	–	0.42 (0.42-0.42)	0.41 (0.40-0.41)	0.42 (0.42-0.42)	–	<.001	–
ICU length of stay	0.15 (0.15-0.15)	–	0.23 (0.23-0.23)	0.21 (0.21-0.21)	0.22 (0.22-0.22)	–	<.001	–
Clinical end point†								
Mortality	–	0.67 (0.67-0.68)	0.69 (0.68-0.70)	0.69 (0.68-0.70)	0.69 (0.69-0.70)	0.91 (0.91-0.92)	<.001	<.001
Acute kidney injury	–	0.67 (0.67-0.68)	0.76 (0.75-0.77)	0.76 (0.76-0.77)	0.74 (0.73-0.75)	0.84 (0.83-0.86)	<.001	<.001
Postoperative transfusion	–	0.71 (0.71-0.72)	0.73 (0.73-0.73)	0.71 (0.71-0.71)	0.73 (0.73-0.74)	–	<.001	–
Reoperation	–	0.81 (0.80-0.81)	0.8 (0.79-0.80)	0.80 (0.80-0.80)	0.79 (0.79-0.80)	0.76 (0.76-0.77)	.99	<.001

Values are presented as mean (95% CI). *GBM*, Gradient boosted machine; *RF*, random forest; *XGBoost*, extreme gradient boosting; *STS*, Society of Thoracic Surgeons risk score; *ICU*, intensive care unit.

∗ Models with continuous output were evaluated using the coefficient of determination (R^2^).

† Binary classifiers were assessed with the area under the receiver operating characteristic.

**Figure 2:**
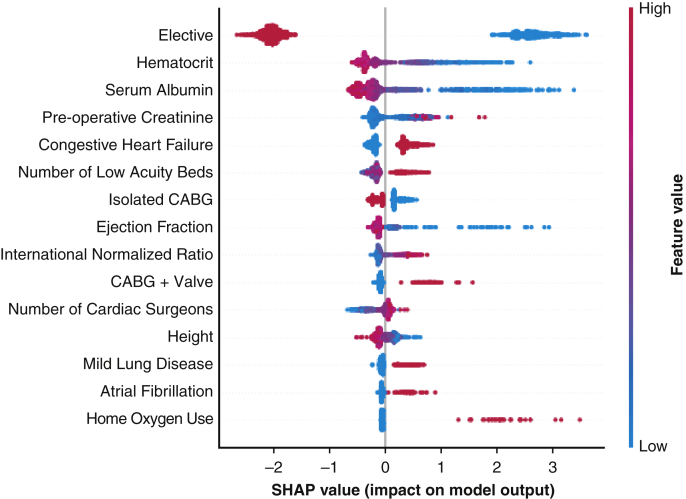
Interpretation of gradient boosted machine (*GBM*)-based model for prediction of length of stay (*LOS*) (days) using SHapley summary plots. The *y*-axis is ordered by increasing feature importance, and the *x*-axis is the marginal effect of each parameter on predicted LOS. Red dots show the influence of high feature values on predicted LOS, whereas blue dots show the influence of low feature values. *CABG*, Coronary artery bypass grafting.

The GBM model was interpreted using SHapley summary plots, and the most salient predictors of LOS were ranked by their relative importance (*y*-axis). Figure 2 depicts how high (*red dot*) and low (*blue dot*) feature values corresponded to a change in LOS prediction. Elective admission had the highest feature importance and was associated with significantly decreased LOS. In addition, we found decreased hematocrit and serum albumin levels to increase the estimated LOS. Certain procedures, such as concomitant CABG/valve and multivalve operations, were found to confer longer LOS. Notably, an increased number of floor beds conferred greater estimated LOS (Figure 2).

**Figure 3:**
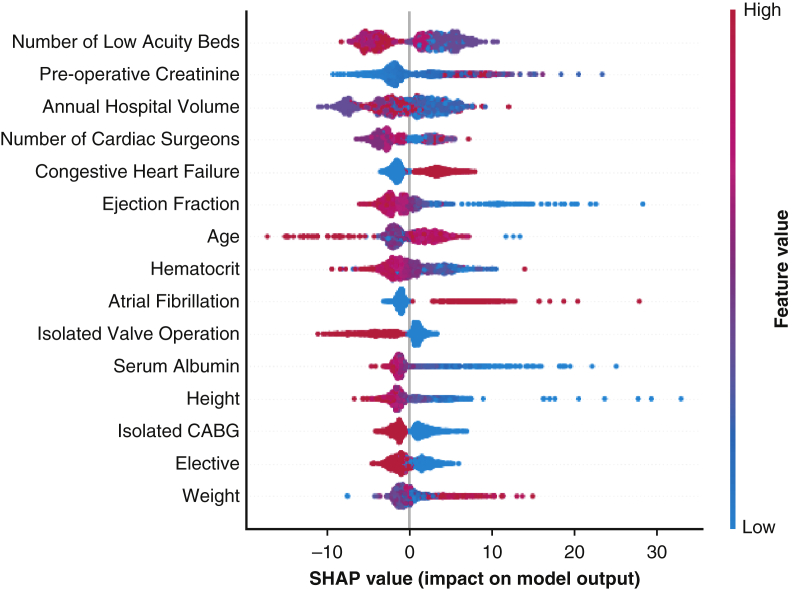
Interpretation of gradient boosted machine (*GBM*)-based model for prediction of intensive care unit length of stay (*ICU LOS*) (hours) using SHapley summary plots. The *y*-axis is ordered by increasing feature importance, and the *x*-axis is the marginal effect of each parameter on predicted ICU LOS. Red dots show the influence of high feature values on predicted ICU LOS, whereas blue dots show the influence of low feature values. *CABG*, Coronary artery bypass grafting.

In the prediction of ICU LOS, the GBM model demonstrated significantly increased cross-validated *R*^2^ compared with linear regression (0.23 vs 0.15; *P* < .001). However, in the validation dataset, the XGBoost model demonstrated the highest performance (Table 4). Decreased preoperative creatinine level, low ejection fraction, and preexisting congestive heart failure were associated with greater predicted ICU LOS. Notably, increased annual hospital volume and a higher number of low-acuity beds were associated with lower estimated ICU LOS (Figure 3).

### Clinical Outcomes

The GBM, RF, and XGBoost models outperformed logistic regression in the prediction of 30-day mortality (AUC, 0.69 vs 0.67; *P* < .001). Furthermore, the GBM and RF models outperformed logistic regression and XGBoost in the prediction of AKI (Table 3). Whereas postoperative blood transfusion was best predicted by GBM and XGBoost, all modeling strategies displayed similar discrimination in the estimation of reoperation (Table 3). The STS risk score for 30-day mortality and AKI outperformed ML models. However, ML displayed greater discrimination than the STS model in the prediction of reoperation (Table 3). These comparisons were consistent when evaluating the Brier score for each model (Tables E4 and E5).

## Discussion

Reliable estimation of hospitalization duration remains a challenge for surgeons and administrators alike. The present study developed several parsimonious ML models to develop a readily useful prediction instrument for LOS (Video 1). This work entails one of the largest applications of ML to discretely model LOS using a multicenter, academic dataset. Compared with linear and logistic regression, we found ML algorithms to exhibit higher performance for prognostication of LOS, 30-day mortality, AKI, postoperative transfusion and ICU LOS. Using autonomous techniques, we identified several key predictors of increased resource use, including existing comorbidities, decreased preoperative hematocrit and serum albumin levels. And finally, we noted a significant influence of hospital characteristics on ICU LOS, suggesting the need for incorporation of center-specific characteristics in predictive tools.

Several clinical characteristics, including preoperative anemia, renal dysfunction, and operative complexity, were associated with increased overall and ICU LOS. These findings are expected because laboratory values such as hematocrit level, international normalized ratio, creatinine level, and albumin level are incorporated in virtually every clinical risk score calculator.^8^ Moreover, these clinical factors influence the development of postoperative complications, including pneumonia and AKI, which are drivers of hospital LOS and costs.^8^^,^^12^^,^^17^^,^^23^ SHapley interpretation revealed that more complex operations were associated with greater LOS. The relatively higher incidence of complications in the setting of complex cardiac surgery, such as pacemaker placement, need for blood transfusion, and a greater need for ICU-level care, may explain this observation. Taken together, our findings validate the utilization of ML methods to reduce bias, enhance external validity, and autonomously select features associated with increasing LOS. Furthermore, our results demonstrate that during times of limited hospital capacity, clinical characteristics such as organ dysfunction and operative complexity should be considered when predicting hospitalization duration.^24^

In addition to patient factors, we found certain hospital structural characteristics to influence ICU LOS. For example, increasing cardiac institutional volume and a greater number of low-acuity beds was associated with reduced ICU LOS. Several factors may contribute to this important finding. Higher institutional cardiac surgery volume may represent greater expertise, the presence of standardized care pathways, and more efficient hospital throughput for these cases. Moreover, greater availability of low-acuity beds may lead to less delay in transitioning out of the ICU when clinical milestones are met.^15^ Consistent with this notion, several prior studies have demonstrated wide variation in hospital practices that may influence LOS, such as expedited discharge after lung resection and CABG.^25^^,^^26^ A nationwide study of minimally invasive esophagectomy in the Netherlands demonstrated great heterogeneity in ICU LOS, pointing to differences in use of early extubation protocols and analgesic modalities as contributing factors.^27^ Investigation at a broader scale is necessary to confirm the generalizability of our findings and to identify modifiable practice patterns that increase LOS.

In the present work, ML models exhibited superior accuracy in the prognostication of overall and ICU length of stay, compared with linear regression. A single-center study similarly compared linear regression and artificial neural networks, finding the latter to have enhanced LOS prediction for patients undergoing isolated CABG.^28^ Furthermore, LaFaro and colleagues^29^ used a sample of 185 patients undergoing cardiac surgery to show that artificial neural networks yield more accurate estimates of ICU LOS compared with linear regression. The improved performance of ML models is likely attributable to their ability to capture nonlinear interactions between covariates and outcomes of interest. Although the decision-tree structure evaluates such interactions autonomously, linear regression models can only accommodate explicitly included interaction terms, making the development of an equivalent model cumbersome and more prone to bias. Our findings are in congruence with the growing body of literature, which demonstrates increased performance of ML models in the clinical setting.^16^, ^17^, ^18^ Thus, ML algorithms should be considered as a viable and potentially superior alternative modeling approach in surgical care applications.

Although ML methods outperformed linear strategies for prediction of reoperation, the STS models outperformed ML for 30-day mortality and AKI. This observation is most attributable to the large sample used to derive the STS risk scores as well as the incorporation of more than 100 data fields.^14^ Nonetheless, the STS models are limited to operations either involving CABG or single-valve replacement, and do not provide risk estimates for aortic surgery or multivalve procedures. Such operations present a more heterogeneous risk profile and may reduce the performance of predictive models. We opted to include such operations in our modeling attempts to develop a tool that accurately reflects the case-mix at our 5 academic institutions. Indeed, procedures not accounted for by the STS comprised approximately 5% of our study cohort. Regardless, ML approaches are gradually being incorporated into the STS models to provide more bespoke estimates, an effort that will certainly improve risk prediction across cases performed in the United States.

The predictive models developed in the present work have considerable utility in the clinical and administrative settings. Their mode of application is tunable to an institution's needs, and the insights that they provide have the potential to enhance clinical outcomes. A landmark randomized control trial by Shimabukuro and colleagues^30^ found the implementation of ML models to reduce ICU mortality and LOS, demonstrating that such tools can tangibly improve clinical outcomes and decrease resource utilization. Our group has chosen to make the ML models with the greatest R^2^ and AUC available for public use. This online tool may be used by clinicians when evaluating patient risk or by administrators who wish to apply our predictive model at the programmatic level. However, a model that continuously incorporates postoperative events into the estimated LOS would be most pertinent to patient care in the perioperative setting. Further efforts to develop such tools are warranted.

Given the premium placed on low-acuity and ICU beds during the COVID-19 pandemic, hospitals transiently reduced surgical volume. Prachand and colleagues^31^ proposed a widely used framework for triaging medically necessary, time sensitive procedures. It highlighted several key factors, such as operating-room time, estimated LOS, and anticipated blood loss, when determining resource allocation. In the event of significant reduction in operating capacity, the development of algorithms that balance risk associated with delay in operative management as well as estimated resource use may be necessary. Our proposed ML based models may better inform decisions about scheduling and optimizing case-mix to ensure sufficient hospital throughput. With wide availability of ML present and use of few explanatory variables, prospective studies may readily determine the pragmatic influence of such models in optimizing hospital efficiency.

The present study has several limitations. As a multicenter study confined to a group of academic centers, our findings are not generalizable to the cardiac surgical population at large. In addition, although the consortium makes a concerted effort to homogenize practice patterns across participating institutions, certain clinical factors may vary by center and surgeon, such as the threshold for blood transfusion. Transfer status was similarly not captured in the UCCSC and could not be accounted for in our predictive models. Furthermore, despite the relatively large size of the dataset, prospective application of the ML models is required to externally validate their utility. Nonetheless, we used robust statistical methods and a sparse set of autonomously selected variables to enhance the generalizability.

## Conclusions

**Figure 4:**
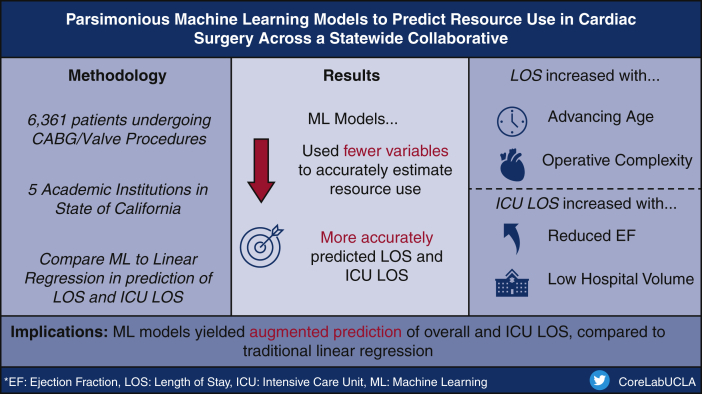
Compared with linear regression, machine learning models exhibited superior performance in the estimation of length of stay following cardiac operations. *CABG*, Coronary artery bypass grafting; *LOS*, length of stay; *ICU LOS*, intensive care unit length of stay; *EF*, ejection fraction.

We have demonstrated the superior performance of ML models in providing accurate predictions for LOS using a multi-institutional, cardiac surgery database. Derived from few variables, such models can estimate resource use and better inform projected hospital census. Leveraging the information derived from machine learning models may be especially useful in reducing the influence of pandemic-related disruptions in cardiac surgical programs (Figure 4).

### Conflict of Interest Statement

Dr Shemin serves as a consultant to the Edwards Lifesciences Advisory Board and as a co-principal investigator on the PARTNER II trial. All other authors reported no conflicts of interest.

The *Journal* policy requires editors and reviewers to disclose conflicts of interest and to decline handling or reviewing manuscripts for which they have a conflict of interest. The editors and reviewers of this article have no conflicts of interest.

## Figures and Tables

**Table E1:** Distribution of missing variables in the entire study cohort, as well as between the derivation and validation cohorts

Parameter	Overall (n = 6316)	Derivation (n = 5028)	Validation (n = 1288)
Age	0	0	0
Elective admission	0	0	0
Female	0	0	0
Height (cm)	2	2	0
Weight (kg)	1	1	0
Ethnicity	0	0	0
Operative type			
Isolated CABG	0	0	0
Isolated valve operation	0	0	0
CABG + valve	0	0	0
Multiple valve	0	0	0
Medical conditions			
Atrial fibrillation	0	0	0
Cancer	0	0	0
Cerebrovascular disease	0	0	0
Mild lung disease	0	0	0
Severe lung disease	0	0	0
Congestive heart failure	0	0	0
Diabetes	0	0	0
Home oxygen	0	0	0
Hypertension	0	0	0
Infectious endocarditis	0	0	0
Intra-aortic balloon pump	0	0	0
Liver disease	0	0	0
Mediastinal radiation	0	0	0
Peripheral vascular disease	0	0	0
Prior myocardial infarction	0	0	0
Syncope	0	0	0
Thoracic aortic disease	0	0	0
Current tobacco use	0	0	0
Laboratory values			
Hematocrit (%)	39	37	2
International normalized ratio	275	232	33
Serum albumin (g/dL)	940	793	147
Preoperative creatinine (mg/dL)	25	22	3
Ejection fraction (%)	198	178	20
Hospital of operation			
Center 1	0	0	0
Center 2	0	0	0
Center 3	0	0	0
Center 4	0	0	0
Center 5	0	0	0

*CABG*, Coronary artery bypass grafting.

**Table E2:** Hyperparameters for each machine learning model developed in the present study. Parameters not mentioned here were set as the default value

Outcome	GBM	RF	XGBoost
Length of stay	{'max_depth': 3, 'max_features': 8, 'n_estimators': 60}	{'max_depth': 6, 'max_features': 7, 'n_estimators': 40}	{'alpha': 1, 'max_depth': 2, 'n_estimators': 30}
ICU length of stay	{'max_depth': 5, 'max_features': 2, 'n_estimators': 70}	{'max_depth': 6, 'max_features': 6, 'n_estimators': 40}	{'alpha': 10, 'max_depth': 3, 'n_estimators': 20}
Mortality	{'max_depth': 2, 'max_features': 7, 'n_estimators': 20}	{'max_depth': 3, 'max_features': 2, 'n_estimators': 10}	{'alpha': 10, 'max_depth': 2, 'n_estimators': 10}
Acute kidney injury	{'max_depth': 3, 'max_features': 10, 'n_estimators': 30}	{'max_depth': 4, 'max_features': 9, 'n_estimators': 50}	{'alpha': 1, 'max_depth': 6, 'n_estimators': 30}
Postoperative transfusion	{'max_depth': 2, 'max_features': 5, 'n_estimators': 10}	{'max_depth': 2, 'max_features': 5, 'n_estimators': 10}	{'alpha': 10, 'max_depth': 2, 'n_estimators': 10}
Reoperation	{'max_depth': 2, 'max_features': 8, 'n_estimators': 10}	{'max_depth': 3, 'max_features': 10, 'n_estimators': 40}	{'alpha': 0.1, 'max_depth': 2, 'n_estimators': 10}

*GBM*, Gradient boosted machine; *RF*, Random forest; *XGBoost*, extreme gradient boosting; *ICU*, intensive care unit.

**Table E3:** Covariates selected for final model development

Selected covariates
Baseline characteristics
Age
Elective operation
Height (cm)
Weight (kg)
Operation type
Isolated CABG
Isolated valve operation
CABG + valve
Multiple valve
Medical conditions
Preoperative atrial fibrillation
Cerebrovascular disease
Mild lung disease
Severe lung disease
Congestive heart failure
Home oxygen
Previous myocardial infarction
Hematocrit
International normalized ratio
Serum albumin
Preoperative creatinine
Ejection fraction
Hospital characteristics
Annual hospital volume
No. of cardiac surgeons on staff
No. of low-acuity beds

*CABG*, Coronary artery bypass grafting.

**Table E4:** Cross-validated Brier scores for binary classifiers

Outcome	Logistic	GBM	RF	XGBoost	STS
Mortality	0.0096 (0.0095-0.0096)	0.0105 (0.0104-0.0107)	0.0095 (0.0095-0.0095)	0.0095 (0.0094-0.0095)	0.0075 (0.0073-0.0077)
Acute kidney injury	0.0146 (0.0145-0.0146)	0.0161 (0.0159-0.0163)	0.0144 (0.0143-0.0144)	0.0145 (0.0145-0.0146)	0.0175 (0.0175-0.0175)
Postoperative transfusion	0.1827 (0.1823-0.1831)	0.1781 (0.1777-0.1784)	0.1894 (0.1892-0.1896)	0.1755 (0.1751-0.1759)	–
Reoperation	0.0727 (0.0725-0.0729)	0.076 (0.0759-0.0761)	0.0739 (0.0738-0.0741)	0.0746 (0.0744-0.0747)	0.0603 (0.0601-0.0604)

*GBM*, Gradient boosted machine; *RF*, Random forest. *XGBoost*, extreme gradient boosting; *STS*, Society of Thoracic Surgeon Risk Score.

**Table E5:** Evaluation of Brier score for each predictive model using data from the validation cohort

Outcome	Logistic	GBM	RF	XGBoost	STS
Mortality	0.0069	0.0083	0.0069	0.0067	0.0070
Acute kidney injury	0.0168	0.0165	0.0162	0.0175	0.0170
Postoperative transfusion	0.1733	0.1708	0.1715	0.1751	–
Reoperation	0.0586	0.0605	0.0594	0.0612	0.0597

*GBM*, Gradient boosted machine; *RF*, Random forest; *XGBoost*, extreme gradient boosting; *STS*, Society of Thoracic Surgeon Risk Score.

## Supplementary Data

Video 1 Authors briefly discuss the performance of machine learning models in the prediction of resource use following cardiac operations. Video available at: https://www.jtcvs.org/article/S2666-2736(22)00171-1/fulltext.
