# Supplementary figures and images for: Parsimonious machine learning models to predict resource use in cardiac surgery across a statewide collaborative
Source: JTCVS Open. 2022 Apr 20;11:214–28. doi: 10.1016/j.xjon.2022.04.017 (PMC9510828; doi:10.1016/j.xjon.2022.04.017)

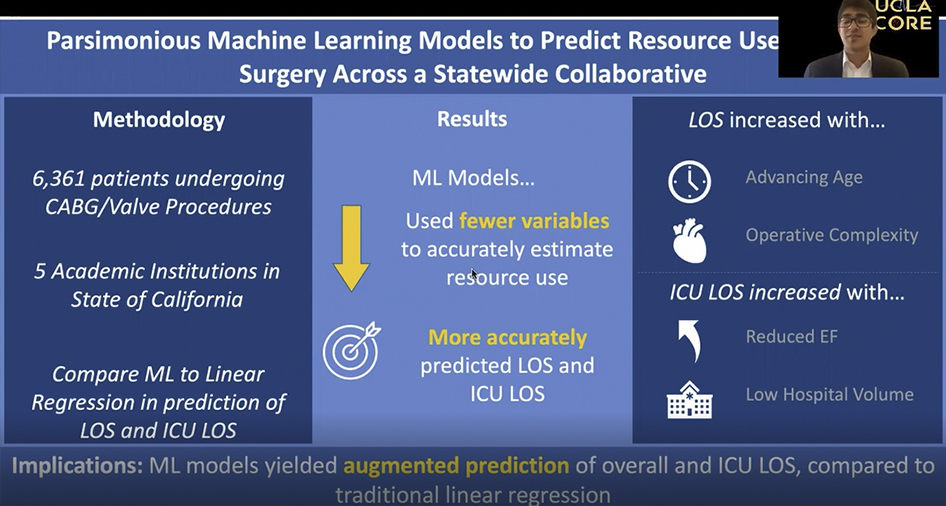

Supplement: Video 1 — Authors briefly discuss the performance of machine learning models in the prediction of resource use following cardiac operations. Video available at: https://www.jtcvs.org/article/S2666-2736(22)00171-1/fulltext. [file fx3.jpg]
